# Extracellular vesicles modulate skin aging biomarkers in a 3D reconstructed full-thickness skin model

**DOI:** 10.3389/fcell.2026.1784998

**Published:** 2026-03-06

**Authors:** Yao Teng, Elias Bou Samra, Sarah Girardeau-Hubert, Richard J. Betts, Franck Juchaux, Xavier Marat, Benedicte Fallou, Lingyan Zhong, Rodrigo De Vecchi, Nan Huang, Qian Zheng, Yu Gao, Daniel C. Roy, Ping Wang

**Affiliations:** 1 Advanced Research, L'Oréal Research & Innovation, Shanghai, China; 2 Advanced Research, L'Oréal Research & Innovation, Aulnay-sous-Bois, France; 3 Advanced Research, L'Oréal Research & Innovation, Biopolis, Singapore; 4 EPISKIN, L'Oréal Research & Innovation, Lyon, France; 5 Advanced Research, L'Oréal Research & Innovation, Clark, NJ, United States

**Keywords:** extracellular vesicle (EV), mesenchymal stem cell (MSC), omics, reconstructed skin model, skin regeneration

## Abstract

Extracellular vesicles (EVs) are lipid-enveloped nanovesicles rich in microRNAs, proteins and lipids, that serve as potent mediators of intercellular communication. While EVs have demonstrated pro-regenerative potential in 2D and preclinical models, their impact on skin regeneration and aging processes in 3D reconstructed skin models has remained less explored. In this study, EVs from adipose-derived stem cells and umbilical cord-derived mesenchymal stem cells (UC-MSCs) were evaluated using both 2D primary skin cells and 3D full-thickness reconstructed skin models. EVs stimulated fibroblast and keratinocyte proliferation, increased epidermal thickness, and enhanced the presence of collagen IV in the dermal-epidermal junction (DEJ) and fibrillin 1 in the extracellular matrix. Bulk transcriptomic analysis of the 3D reconstructed skin revealed gene expression profiles impacted by the addition of EVs. Additionally, miRNA-seq and proteomics of extracellular vesicle contents revealed miRNAs and proteins that may be drivers of the biological activities observed in 3D models, suggesting EVs activate processes associated with skin regeneration. This holistic approach demonstrated that EVs previously linked to pro-regenerative behaviors also modulate biomarkers associated with cutaneous aging in full-thickness 3D reconstructed models. This work not only provides mechanistic insights but also paves the way for the development of next-generation regenerative skincare active ingredients.

## 1 Introduction

Bio-based materials, including platelet rich plasma (PRP) (Alsousou et al., 2013), extracellular vesicles (EVs) (Lee and Kim, 2021), stromal vascular fraction (SVF) (Bora and Majumdar, 2017) and a range of isolated growth factors (Ren et al., 2020), are emerging technologies for tissue regeneration (Crowley et al., 2021), acting to promote local cell proliferation, differentiation, migration, angiogenesis, matrix deposition and remodeling (Iismaa et al., 2018; Shi H. et al., 2021; Brunet et al., 2023; Davies et al., 2023). Among these materials, EVs are natural nanovesicles produced by cells, in part, to deliver messages as a means of cell-to-cell communication (Liang et al., 2014). The ability of EVs to influence intercellular communication heralds them as a promising therapeutic approach for the treatment of tissue aging, regenerative aesthetics, and scar and burns treatment either as a standalone clinical procedure or an adjunct to existing treatments (Nagelkerke et al., 2021; Vyas et al., 2023; Zheng et al., 2023).

Extracellular vesicles consist of a phospholipid bilayer, and can carry a diverse array bioactive components including proteins, nucleic acids, and lipids (Berumen Sánchez et al., 2021). The cargo and subsequent bioactivity of EVs is a function of the cell from which they are derived; moreover, EVs have distinct characteristics compared to their parental cells that are favorable for clinical use, such as the inability to self-replicate, reduced immunogenicity, and improved transportability and storage (Nagelkerke et al., 2021; Zhao H. et al., 2023; Zheng et al., 2023). There are several examples in recent literature demonstrating the regenerative potential of EVs from various cell sources. Mesenchymal stem cell (MSC)-derived EVs have been linked to expedited skin wound healing through their involvement in inflammation regulation, angiogenesis, cell proliferation, ECM remodeling, and through mitigating oxidative stress in the wound microenvironment (Dorronsoro et al., 2021; Quiñones-Vico et al., 2021; Chen et al., 2023; Zhang et al., 2025). For instance, umbilical cord MSC-derived EVs enhanced skin cell proliferation and viability *in vitro* and promoted skin regeneration in rat burn models *in vivo* by activating both the AKT and Wnt4/β-catenin signaling pathways (Zhang et al., 2015). Additionally, EVs derived from human dermal fibroblasts (HDFs) and bone marrow mesenchymal stem cells (BM-MSCs) have shown promise for reversing skin aging in mouse models, increasing procollagen type I levels and decreasing the expression of the matrix-degrading enzyme MMP-1 (Hu et al., 2019). Dorronsoro and colleagues demonstrated that MSC-EVs reduced SA-β-gal + senescent murine embryonic fibroblasts (MEFs) and IMR-90 fibroblasts numbers in 2D culture, and suppressed senescence *in vivo* while extending health span within a p16INK4a-luciferase expression mouse model (Dorronsoro et al., 2021). EVs isolated from induced pluripotent stem cells (iPSCs) stimulated the proliferation and migration of human dermal fibroblasts and protected against UVB irradiation-induced damage and overexpression of matrix-degrading enzymes (MMP-1/3) (Oh et al., 2018). EVs sourced from human platelets have recently been implicated for a range of regenerative applications including wound healing and tendon repair (Shi A. et al., 2021; Graça et al., 2022). Collectively, these studies demonstrate how EVs from a diverse set of sources can promote cell behaviors linked to skin regeneration.

Despite increasing evidence of EV bioactivity through *in vitro* and pre-clinical models, clinical data is only recently emerging demonstrating that EV-containing formulas are beneficial for aesthetics and skin regeneration applications (Mehta et al., 2023; Park et al., 2023). A 12-week double-blind trial examining the safety and efficacy of ADSC-EVs combined with fractional CO_2_ laser treatment for acne scars found the side of the face receiving ADSC-EVs showed a more pronounced reduction in acne scar scoring, less erythema and shorter downtime compared to the vehicle-treated side (Kwon et al., 2020). Another study demonstrated the safety and efficacy of a topical platelet-derived exosome product serum in a single-arm, longitudinal study aimed at reducing redness and improving skin health (Proffer et al., 2022). Further investigation is needed to fully elucidate the safety profile of exosomes in dermatological applications and determine optimal concentration and delivery methods for various skin conditions. In addition to the limited published reports, several clinical studies are ongoing to assess EVs for regenerative and aesthetic outcomes including as ingredients to support wound healing (ClinicalTrials.gov: NCT05475418) and as injectables to slow the aging process (ClinicalTrials.gov: NCT05813379). Despite recent advancements and acceleration into clinical evaluation, no EV products have received full approval by the US Food and Drug Administration.

While there is increasing evidence of the positive impact of mammalian EVs on skin diseases and skin regeneration, there remains considerable gaps in knowledge of EV efficacy and mechanisms of action. Traditional 2D cell assays lack crucial elements of cell-cell interactions and are unable to examine endpoints related to three-dimension structure, while animal studies struggle to replicate human skin physiology. Available clinical data on EVs is minimal and comes with challenges in decoding mechanisms. *In vitro* 3D models employing human cells offer several advantages in that they are reproducible, allow for the study of human cells in a 3D microenvironment, and can be sampled and processed for a variety of biomarkers to gain mechanistic insights. Within the present study, 3D organotypic skin models reconstructed from primary human cells were utilized to circumvent the limitations of many existing published approaches, with the aim to assess EV potential in promoting skin regeneration. Adipose-derived stem cell EVs and umbilical cord-derived mesenchymal stem cell EVs were evaluated in this study, as each EV source has previously been reported to demonstrate a regenerative signature (Pittenger et al., 2019). Extracellular vesicles from ADSC and UC-MSC were examined to conduct extensive efficacy evaluation across key markers of skin regeneration and aging, and via RNA-seq analysis. Furthermore, miRNA-seq and proteomics approaches were used to gain insights into ADSC EV and UC-MSC EV contents and biological properties.

## 2 Materials and methods

### 2.1 EV isolation and characterization

Extracellular vesicles sourced from ADSCs and UC-MSCs were acquired for this work due to their previously established regulatory effects on skin cells. The studies conducted herein were solely for research, knowledge, and discovery purposes and do not imply any commercial interest in these specific EVs. The EVs used in this study were produced by EchoBiotech (Beijing, China), using commercial human cell lines UC-MSC (ATCC #PCS-500-010) and ADSC (ATCC #PCS-500-011). The cells obtained were expanded according to the manufacturer’s standardized protocols. After reaching 80% confluence, third-passage ADSCs and UC-MSCs were subjected to a phosphate-buffered saline (PBS) rinse and cultured in serum-free medium (Yocon, China) for 48 h. The conditioned medium was collected and centrifuged to isolate and purify EVs following the constructed protocol (Fan et al., 2022; Yu D. et al., 2023). Briefly, the conditioned medium was centrifuged at 3,000 *g* for 30 min at 4 °C, followed by filtering with 0.45 μm and 0.22 μm membranes (Steritop TM, Millipore) to remove the remaining cells and cellular debris. Finally, EVs were isolated by size fractionation and concentrated by centrifugation at 3,000 × g for 10 min at room temperature (RT) using an ultra-clear tube (Millipore) with a molecular weight cutoff of 100 kDa. EVs were stored at −80 °C for the following experiments.

The size and particle count of ADSC EVs and UC-MSC EVs were determined by nanoparticle tracking analysis (NTA) with Zeta View PMX 110 (Particle Metrix, Germany) equipped with a 405 nm laser and CMOS camera. For analysis, EV suspensions were diluted in 1 mL PBS to reach the instrument detection range between 1 × 10^7^ particles/mL and 1 × 10^9^ particles/mL. Data acquisition was performed through a 60 s video at 30 frames/s. Particle Brownian motion was subsequently analyzed using ZetaView software (v8.02.28) to calculate the final size profiles and particle counts. The final particle concentration of the original stock was calculated by multiplying the instrument’s readout by the respective dilution factors. All measurements were performed in triplicate to ensure statistical reliability.

Cryo-TEM (Talos F200C G2) was used for sample observation. For preparation, a 3 μL droplet of each sample was applied to a holey carbon grid, blotted and then plunge-frozen into precooled liquid ethane using a Leica EM GP Cryo preparation chamber (Leica). The sample was then embedded in a thin layer of vitreous ice, ensuring protection from radiation damage and preservation of the vesicle structures. Imaging was performed at a 200 kV acceleration voltage.

For Western blot analysis, total protein was first quantified via BCA Protein Assay Kit (Thermo Fisher Scientific). Samples were then denatured in sodium dodecyl sulfonate (SDS) buffer and heated at 95 °C. An equivalent amount of protein (30 µg) was added to each lane and subjected to 10% SDS-polyacrylamide gel electrophoresis (SDS-PAGE). Antibodies utilized included TSG101 (ab125011, Abcam), HSP70 (ab181606, Abcam), CD63 (sc-5275, Santa Cruz), and calnexin (10427-2, Proteintech).

### 2.2 Cell culture and fibroblast proliferation assay

Normal human skin tissue specimens were acquired through mammary reduction procedures with ethical approval (see details in the Ethical Section). Normal human fibroblasts (NHF) were subsequently isolated from the dermis and cultured in high-glucose DMEM (Invitrogen) supplemented with 10% fetal bovine serum (FBS). Cells were maintained in a humidified atmosphere at 37 °C with 5% CO2 and underwent passaging every 3–4 days, utilizing cells from passages 3-5 for experimental purposes.

The NHF cells were harvested and seeded into 96-well plates at a density of 2,000 cells per well in medium containing 10% FBS. After 24 h incubation, the medium was replaced with one containing 2% FBS, with or without the designated treatment. A positive control group was also included, which continued to receive 10% FBS. Cells under various treatment conditions were then maintained for an additional 3 days. For assessing cell proliferation, the culture medium was aspirated, and the CyQuant kit (Invitrogen, C35006) was introduced to the cells, followed by incubation at 37 °C for 1 h in accordance with the manufacturer’s instructions. Subsequently, the results were measured using a plate reader (SpectraMax, Molecular Devices). The cell stimulation range was normalized to 100% for cells treated with 10% FBS versus 2% FBS, and the stimulation ratio of EV-treated groups was calculated relative to this baseline.

### 2.3 Cellular uptake of EVs

Extracellular vesicles were labeled using the CM-Dil red fluorescent membrane linker dye (C7000, Invitrogen), following the manufacturer’s guidelines. In brief, 200 μg of EVs suspended in 500 μL of PBS were mixed with 5 μL of CM-Dil stock solution (1 mg/mL) and incubated at 37 °C for 5 min, followed by a 15-min incubation at 4 °C. Then, unbound CM-Dil was removed by thoroughly washing the EVs with PBS via ultrafiltration centrifugation. NHF cells were then exposed to CM-Dil-labeled EVs at a concentration of 100 μg/mL for a duration of 12 h. Subsequently, NHF cells underwent three washes with PBS, were fixed in 4% paraformaldehyde, and were stained with DAPI. Finally, cellular uptake of the labeled EVs was observed using a Zeiss Confocal LSM 710 microscope. To assess uptake efficiency, the percentage of the fibroblast population with perinuclear EV co-localization was quantified by calculating the ratio of EV-positive cells to the total number of DAPI-stained nuclei across multiple representative fields.

### 2.4 Full-thickness skin equivalent model

The reconstructed skin models employed in this study (T-skin^TM^ from EPISKIN) were produced in a 6-well plate format with a surface area of 4 cm^2^ per unit following established procedures (Bataillon et al., 2019). Briefly, the dermal compartment was formed using a blend of collagen and dermal fibroblasts. Normal human keratinocytes (NHK) were subsequently seeded onto this layer on day 5. These models were incubated in a submerged environment using appropriate culture media for a duration of 6 days. Following this period, the skin tissue was transitioned to an air-liquid interface culture for an additional 7 days before being harvested.

### 2.5 OCT imaging and quantitative processing

To obtain cross-sectional views of the full-thickness skin model, optical coherence tomography (OCT, Thorlabs, Newton, NJ, United States) was utilized, featuring an axial resolution of 8 μm (center wavelength of 930 nm and penetration depth of 1.5 mm). Optical imaging was performed using a 3D scan volume measuring 9.42 × 9.42 × 1.5 mm, comprising 512 X-Z slices for each sample. The thickness of the epidermal layer was quantified by ImageJ (NIH, Bethesda, MD, United States), based on optical images obtained via OCT. The average thickness was determined by dividing the area of the whole epidermal layer by its corresponding length.

### 2.6 Histology and immunofluorescent staining

The full thickness skin model was punched out with a 10 mm-diameter punch for histological analysis and immunofluorescence staining. Punched samples were bisected, with one-half of each sample fixed in 10% formalin and subsequently processed for paraffin embedding. The embedded samples were sectioned into 5 μm-thick slices using a microtome (Leica RM2255) and stained with hematoxylin and eosin (H&E) for histological analysis. For immunofluorescent staining, skin samples were embedded in Optical Cutting Temperature compound (O.C.T) for cryo-sectioning. The 7 μm-thick sections were air-dried for 1 h at room temperature and then fixed in cold acetone for 5 min. Following two washes with DPBS, the sections were blocked with 0.2% BSA in DPBS for an additional 10 min. Primary antibodies, including Ki67 (M7240, mouse, 1:100, Dako), Collagen IV (MAB1430, mouse, 1:100, Sigma), and Fibrillin 1 (1,405-01, mouse, 1:50, Southern Biotech), were diluted in DPBS containing 1% donkey serum and incubated at room temperature for 2 h in a humid chamber. Afterward, the sections were washed with DPBS three times. Subsequently, secondary antibodies (donkey anti-mouse Alexa Fluor 488, A21202, 1:300, Invitrogen) were diluted in DPBS containing 1% donkey serum and incubated for 1 h at room temperature, followed by three DPBS washes. Finally, the sections were stained with DAPI (D1306, 1:10,000, Invitrogen) and washed with DPBS. Imaging was conducted using a Nikon Eclipse Ti fluorescence microscope. All the fluorescent images were processed using ImageJ software.

### 2.7 Bulk transcriptomic analysis

For full-thickness skin model bulk RNA-seq, 5-6 individual replicates were used to represent models exposed to various conditions. Total RNA was extracted using the Qiagen RNeasy Mini Kit. Library construction and sequencing were outsourced to Shanghai Biotechnology Corporation (Shanghai, China) following their protocol as reported (Chen et al., 2022; Wu et al., 2023; Yang et al., 2023), with strand-specific libraries prepared using the TruSeq Stranded Total RNA Sample Preparation kit (Illumina, United States). Purified libraries underwent quantification with the Qubit 2.0 Fluorometer (Life Technologies, United States) and validation by the Agilent 2,100 bioanalyzer (Agilent Technologies, United States). Sequencing was performed on the Illumina Nova Seq 6,000. Clean reads were then mapped to the human reference genome, and the uniquely mapped gene fragments were quantified using Stringtie (version 1.3.0). Expression levels were normalized via the Trimmed Mean of M values (TMM) method and converted to FPKM (Fragments Per Kilobase of transcript per Million mapped fragments). Differentially expressed genes (DEGs) were identified using EdgeR package (version 3.30.3) with |FoldChange| ≥ 2 and Q-values <0.05. Pathway enrichment analysis was conducted using the ClusterProfiler package (version 3.16.1) in the R programming language (version 3.6.2).

### 2.8 MicroRNA sequencing and differentially expressed miRNA analysis

MiRNA sequencing was performed by Echo Biotech (Beijing, China) following established protocols (Wang et al., 2021; Luo et al., 2023). Initially, total RNA was extracted from the samples using the Qiagen exoRNeasy Midi/Max Kit in accordance with the manufacturer’s guidelines. The extracted total RNA underwent assessment using the Agilent 2,100 Bioanalyzer (Agilent Technologies, United States). After quantification of total RNA, the sequencing RNA library was prepared using the Small RNA Sample Pre Kit. This comprehensive process included adding 3′-end and 5′-end linkers, reverse transcription, amplification, cDNA library size selection, and purification. Preliminary quantification was performed using Qubit 2.0, and the library’s insert size was measured with the Agilent 2,100 instrument. Sequencing was carried out on the Illumina Hiseq 2,500 platform, meeting the specified criteria for effective concentration and the required volume of offline data. Data collection and real-time analysis were managed using Illumina’s data collection software. Differentially expressed miRNAs between groups were analyzed using the DESeq R package (version 1.8.3), with significance criteria set at a corrected P-value of 0.05 and a minimum 1.5-fold change. The analysis of Gene Ontology (GO) and Kyoto Encyclopedia of Genes and Genomes (KEGG) pathway enrichment for the target genes of miRNAs was conducted subsequently.

### 2.9 Proteomics analysis of EVs

To measure the protein content of EVs, label-free quantitative proteomics analysis was carried out following established protocols (Mao et al., 2021; Yu Y. et al., 2023). Briefly, EVs were lysed and centrifuged with the supernatant collected, followed by protein reduction with dithiothreitol and alkylated with iodoacetamide to extract the protein. Then, samples were mixed with acetone and centrifuged, with the precipitate collected. The protein quality of these samples was determined using the BCA Protein Assay Kit (Thermo Fisher Scientific). Next, the proteins were digested overnight with trypsin, and the resulting digested peptides were desalted using a C18 column. These peptides were dissolved in mobile phase A (0.1% (v/v) formic acid solution) and separated using a NanoElute ultra-high-performance liquid phase system, passing from a C18 trap column to a C18 analytical column. Mobile phase B consisted of an acetonitrile solution containing 0.1% formic acid. The peptides were subjected to a nanospray ionization (NSI) source followed by tandem mass spectrometry (MS/MS) using Q ExactiveTM Plus (Thermo Fisher Scientific) coupled online to the UPLC. Raw MS data were searched against the UniProt database (http://www.uniprot.org). Carbamidomethyl was specified as a fixed modification, while oxidation of methionine (M) and acetylation of the N-terminus were specified as variable modifications. Proteins were considered identified if they contained at least one unique peptide with a false discovery rate (FDR) of no more than 0.01. Gene Ontology (GO) analysis was performed, and the COG and KEGG databases were used for protein family and pathway annotation.

### 2.10 Statistical analysis

Statistical analysis of all data was performed using GraphPad Prism, and the results are presented as mean ± standard deviation, unless otherwise specified. Comparisons between groups were made with ANOVA followed with *post hoc* Dunnett’s test. Significant differences were indicated by asterisks, with *, ** and *** denoted significant differences with p-values less than 0.05, 0.01 and 0.001, respectively.

## 3 Results

### 3.1 Characterization of EVs isolated from ADSCs and UC-MSCs

**FIGURE 1:**
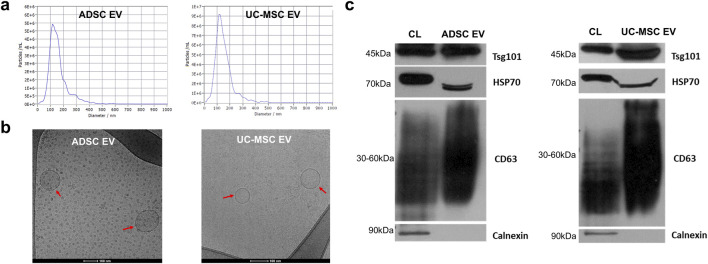
Characterization of ADSC EVs and UC-MSC EVs. **(a)** Size and concentration measurement by NTA; **(b)** Morphology visualization with Cryo-TEM with EVs indicated by red arrows (scale bar: 100 nm); **(c)** EV related biomarkers in the EV groups and cell lysate (CL) detected with Western blot, including positive markers TSG101, HSP70, CD63 and negative marker calnexin.

The EVs obtained from ADSC and UC-MSC cell supernatants underwent characterization in accordance with the MISEV2023 guidelines (Welsh et al., 2024), with the detailed operational procedures outlined in Section 2.1. Nanoparticle tracking analysis (NTA) was employed to determine the size of the EVs, showing the average size of the ADSC EVs was 125 ± 5 nm, and it was 130 ± 7 nm for the UC-MSC EVs, both predominantly falling within a range of 60–200 nm (Figure 1a) and consistent with the previous reports (Xu et al., 2020). Additionally, transmission electronic microscopy (TEM) was utilized to visualize the morphology of the EVs, revealing that both ADSC and UC-MSC EVs exhibited round and cup-shaped structures. These EVs exhibited intact membrane structures and a similar size range of approximately 100–200 nm in diameter as detected via NTA (Figure 1b). To further validate the EV identity, Western blotting was used to confirm the presence of a select EV protein markers, including TSG101, HSP70 and CD63. The results demonstrated the ADSC EVs and UC-MSC EVs expressed positive markers TSG101, HSP70, and CD63 which was also confirmed by the expression in the cell lysate (CL) group. The negative marker calnexin, which should be expressed in the cell lysate, was not expressed in the ADSC EVs and UC-MSC EVs (Figure 1c). The presence of these three positive markers along with the absence of the negative marker calnexin confirms the EV identity.

### 3.2 ADSC EV and UC-MSC EV effect on 2D fibroblasts

**FIGURE 2:**
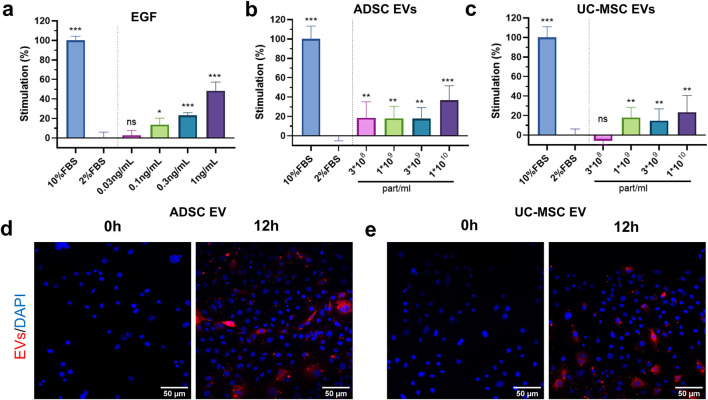
EV effect on 2D NHF cells. **(a–c)** 2D NHF proliferation following addition of EVs; **(d,e)** EV uptake in fibroblasts (Scale bar = 50 µm). One-way ANOVA, *p < 0.05, **p < 0.01, ***p < 0.001 versus 2% FBS group. Data are representative of three independent experiments each performed in triplicate.

Normal human fibroblasts cultured on 2D plates were treated with ADSC EVs and UC-MSC EVs to investigate the impact of EVs on fibroblast proliferation. Epidermal growth factor (EGF), added to a subset of cells as a positive control, stimulated a concentration-dependent increase in cell proliferation (Figure 2a), consistent with previously reported findings (Yu et al., 2012). Extracellular vesicles were applied at final concentrations ranging from 3 × 10^8^ particles/mL to 1 × 10^10^ particles/mL. The results revealed a significant enhancement in fibroblast proliferation compared to the negative control (2% FBS) following ADSC EV addition at all concentrations tested. Notably, treatment with 1 × 10^10^ particles/mL ADSC EVs led to 37% increase in cell proliferation, with an approximately 18% increase observed at lower test concentrations relative to controls (Figure 2b). UC-MSC EVs stimulated fibroblast proliferation at concentrations at or above 1 × 10^9^ particles/mL, culminating in a 23% increase in fibroblast proliferation at the highest concentration tested (1 × 10^10^ particles/mL). Unlike ADSC EVs, 3 × 10^8^ particles/mL of UC-MSC EVs did not yield an increase in cell proliferation (Figure 2c).

To visualize whether EVs are internalized by fibroblasts, the EVs were labeled with the lipophilic fluorescent dye, DiL, and incubated with the fibroblasts for up to 12 h. Images captured at different time points revealed the uptake of EVs by fibroblasts, with both ADSC and UC-MSC EVs being internalized by cells at 12 h (Figures 2d,e). Image analysis of representative fields suggested that approximately 51% and 45% of the fibroblast population exhibited detectable perinuclear co-localization with ADSC-EVs and UC-MSC-EVs, respectively. This provided qualitative and semi-quantitative evidence of EV-cell interaction, providing a mechanistic basis for regenerative responses observed in this work.

### 3.3 ADSC EV and UC-MSC EV effect on 3D skin reconstructed model

**FIGURE 3:**
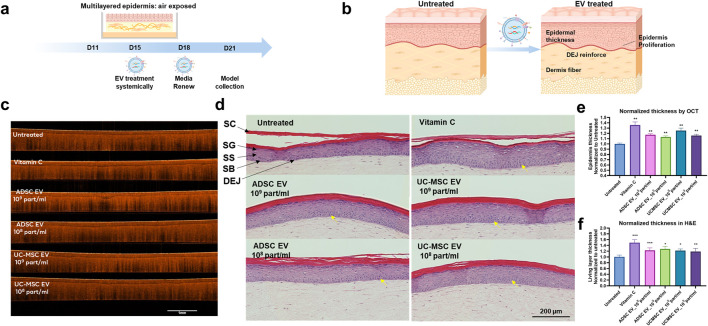
EV effect on epidermal layer in 3D reconstructed skin model. **(a)** Schematic diagram of the experimental flow for T-skin™ model with EV treatment, **(b)** focusing on related key marker changes (created with BioRender.com). **(c)** OCT images of the 3D skin models treated with or without EVs at different concentrations. Scale bar = 1 mm. **(d)** H&E staining of the skin model section treated with or without different EVs conditions. SC: stratum corneum, SG: stratum granulosum, SS: stratum spinosum, SB: stratum basale, DEJ: dermal-epidermal junction. Scale bar = 200 μm. Quantitative results of the **(e)** epidermal thickness based on OCT and **(f)** the thickness of the living cell layers based on H&E images. One-way ANOVA, *p < 0.05, **p < 0.01, ***p < 0.001 versus Untreated group. n = 3, representative of 3 independent experiments each performed in triplicate.

To examine the biological activity of EVs within a reconstructed skin system, the culture media of a full-thickness reconstructed skin model (T-Skin^TM^) was administered ADSC EVs and UC-MSC EVs. In this study, EVs were applied at both a high testing concentration of 1 × 10^9^ particles/mL and a lower concentration of 1 × 10^8^ particles/mL. EV treatment commenced on Day 15 after the model was raised from the air-liquid interface. Culture medium containing the indicated treatment was renewed on Day 18 and incubated an additional 3 days. On Day 21, the models were harvested for various analyses, including OCT imaging, H&E staining, and immunofluorescent staining (Figures 3a,b).

The thickness of the epidermal layer within the skin models was assessed and normalized relative to the untreated group. The application of vitamin C served as a positive control and resulted in a significant increase in the thickness of the full epidermis (Figure 3c). When subjected to treatment with ADSC EVs and UC-MSC EVs, the models exhibited marked enhancements in epidermal thickness at both EV concentrations, as determined by OCT (Figures 3c,e). Since the OCT images encompassed the whole epidermis inclusive of the stratum corneum, H&E staining was performed to visualize distinct layers of the epidermis. The model demonstrated well-defined principal epidermal layers of the stratum basale (SB), stratum spinosum (SS), stratum granulosum (SG), and stratum corneum (SC) (Figure 3d), as previously described (Bataillon et al., 2019). The thickness of the living cell layer was measured, showing a significant increase following EV treatment (Figure 3f). Moreover, as indicated by yellow arrow in Figure 3d, the basal layer of the epidermis exhibited a higher degree of proliferation and polarization, characterized by elongated columnar basal keratinocytes oriented perpendicularly to the dermal-epidermal junction (DEJ), compared to the cuboidal basal keratinocytes observed in the untreated groups.

**FIGURE 4:**
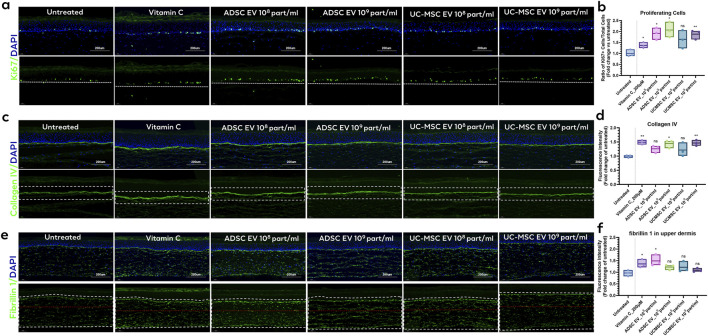
EV effects on aging biomarkers in 3D reconstructed skin model. **(a)** Immunofluorescent images for Ki67 in skin model sections with different EV treatment conditions and **(b)** quantitative results for Ki67-expressing cell ratio. **(c)** Immunofluorescent images for collagen IV in skin model section with or without different EV treatment conditions and **(d)** Semi-quantitative results for collagen IV expression. **(e)** Immunofluorescent images for fibrillin 1 in skin model section with different EV treatment conditions and **(f)** quantitative results for fibrillin 1 expression. One-way ANOVA, *p < 0.05, **p < 0.01, ***p < 0.001 versus Untreated group. n = 3, representative of 3 independent experiments. Scale bar = 200 μm.

Proliferating keratinocytes in the basal layer of the epidermis were visualized via immunostaining with a Ki67 antibody, a key marker of cellular proliferation. The fraction of Ki67-positive keratinocytes in the viable layers of the epidermis was quantified and the Ki67-positive cell ratio in the basal layers was further calculated. Reconstructed skin models demonstrated that ADSC EV treatment at high and low doses resulted in a two-fold increase and a 1.9-fold increase in keratinocytes expressing Ki67, respectively. Treatment with UC-MSC EVs similarly resulted in 1.8 and 1.6-fold increases in Ki67 expression for high and low concentrations, respectively (Figure 4a). The spatiotemporal differentiation process occurring within the epidermis was reflected by the localization of the differentiation biomarker filaggrin, which did not exhibit a significant difference in expression with or without EV treatment (Supplementary Figure S1).

The DEJ biomarker collagen IV, which is part of the intricate network of proteins and proteoglycans that connect the two skin layers and serves important regulatory functions, was also examined post-EV treatment (Bataillon et al., 2019). Collagen IV expression in the T-skin™ model was initially measured following treatment with 200 μM vitamin C, resulting in a significant 49% increase compared to the untreated control. The models treated with EVs derived from both ADSCs and UC-MSCs exhibited significantly elevated collagen IV expression. Collagen IV expression was semi-quantified using image analysis, revealing a 45% increase with high concentrations of ADSC EVs and UC-MSC EVs. For ADSC EVs and UC-MSC EVs tested at low concentrations, there was an increase of 26% that fell short of reaching statistical significance.

Fibrillin 1 was also assessed, which is a structural glycoprotein to which tropoelastin molecules attach, forming elastin fibers in dermal layers. Expression of fibrillin 1 in T-skin™ was measured following treatment with 200 μM vitamin C, resulting in a significant 36% increase compared to the untreated control, consistent with previous reports (Bataillon et al., 2019). Expression of fibrillin 1 showed a 49% increase by ADSC EVs at 10^8^ particles/mL, while the effect size at 10^9^ particles/mL appeared to be less potent (23% increase, p = 0.055 vs. vehicle control). An elevation of fibrillin 1 expression was observed for the UC-MSC EV groups at 10^9^ particles/mL (p = 0.26) and 10^8^ particles/mL (p = 0.20) (Figure 4f); however, these increases were not statistically significant. Notably, fibrillin 1 expression of EV-treated tissues exhibited a more elongated fiber-like structure, compared to the more predominant punctate pattern within control tissues (Figure 4e), although the physiological significance of this remains unclear.

### 3.4 Decoding ADSC and UC-MSC EV mechanisms for skin regeneration with transcriptomic analysis

**FIGURE 5:**
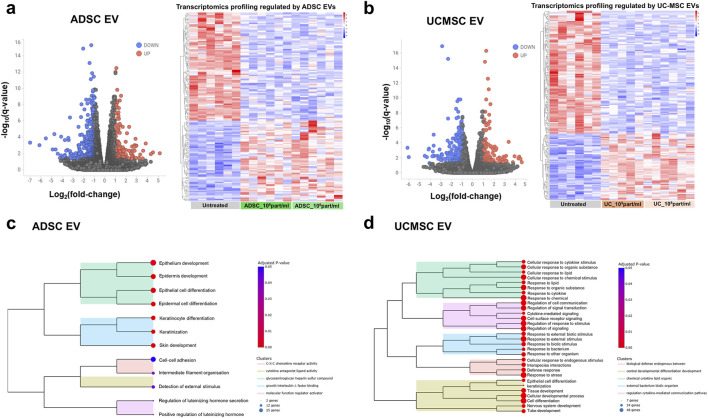
Bulk transcriptomic analysis of a reconstructed skin model after treatment with ADSC EVs and UC-MSC EVs. **(a,b)** Volcano plots and heatmaps showing commonly DEGs regulated by high and low doses of **(a)** ADSC EVs and **(b)** UC-MSC EVs, with 5-8 replicates per group; **(c,d)** Top GO terms enriched among DEGs modulated by **(c)** ADSC EVs and **(d)** UC-MSC EVs, as identified in the GO database.

To elucidate the underlying molecular mechanism in T-skin™ models treated with ADSC EVs and UC-MSC EVs, samples were lysed and processed for bulk transcriptomic analysis. Differential gene expression was assessed by comparing EV-treated groups (at both high and low concentrations) to untreated skin models. Due to the variability observed between individual EV concentrations, even when derived from the same source, a stringent approach was applied: only genes consistently differentially expressed at both high and low EV concentrations were considered to define the robust transcriptomic signature for each EV-treated group (Supplementary Figure S2). This comparative analysis identified a set of differentially expressed genes (DEGs) with Q-value <0.05, among which 142 and 159 genes exhibited at least a two-fold change in expression between EV-treated and control samples under the ADSC EV and UC-MSC EV conditions, respectively (volcano plots, Figures 5a,b). The distinct expression profiles of these DEGs were further visualized through heatmaps of normalized expression values, clearly illustrating the differences between experimental groups (Figures 5a,b).

An overview of the most significantly enriched pathways among the differentially expressed genes is provided in Figures 5c,d and Supplementary Tables S1-S3. In both comparisons, there was a notable enrichment in gene ontology (GO) terms related to skin and epithelial development, and keratinocyte differentiation. These findings are consistent with the results described earlier in the paper Section 3.3. Additionally, we could highlight significant enrichment of GO terms related to cell-cell adhesion. This was accompanied by the modulation of several genes involved in skin junctions, such as increased expression of tight junction proteins CLDN17 and CLDN3, as well as differential expression of CDH2 (N-cadherin) and DLG2, which are key players in cell junction organization and polarity (Supplementary Tables S2-S3). Altogether, these findings suggest that analyzed EVs may enhance skin barrier integrity by influencing pathways critical for cellular adhesion and cytoskeletal organization.

Further pathway enrichment analysis revealed a significant enrichment of GO terms related to cytokine signaling and immune response for the UC-MSC EV condition (Figure 5d; Supplementary Table S3). Notably, several genes central to inflammatory and immune pathways were differentially expressed following UC-MSC EV treatment. For example, IL6 and IL33, both key cytokines involved in mediating inflammatory responses, were downregulated. Chemokines such as CXCL8 and CCL4, which are important for immune cell recruitment and activation, also showed decreased expression. Additionally, genes like CXCR4 and ACKR3, which encode chemokine receptors involved in immune cell trafficking and modulation, were significantly decreased. In contrast, pathway enrichment analysis for the ADSC EV condition did not identify significant enrichment of GO terms related to cytokine signaling or immune response. However, upon manual inspection of the expression data, several genes highlighted in the UC-MSC EV condition also exhibited decreased expression following ADSC EV treatment (Supplementary Tables S2-S3). These genes were likely excluded from the enrichment analysis because their fold-change values fell below the set threshold. Together, these findings suggest that both UC-MSC- and ADSC-derived EVs may play a role in regulating cytokine signaling and immune-related processes in the skin model, although the effect appears more pronounced with UC-MSC EVs.

### 3.5 Micro RNA-seq and proteomics profiling of the ADSC EVs and UC-MSC EVs

**FIGURE 6:**
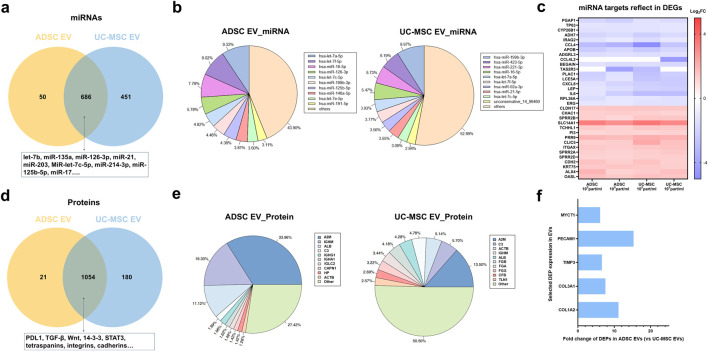
Comprehensive profiling of miRNA and protein cargo in EVs. **(a)** Venn diagram illustrating miRNAs in the two groups of EVs; **(b)** Top abundantly expressed miRNAs identified in each EV types; **(c)** Overlap between predicted target genes of shared miRNAs and DEGs from T-skin™ transcriptomic analysis; **(d)** Identified proteins in the two groups of EVs revealed by Venn plot; **(e)** Top expressed proteins in the two types of EVs; **(f)** Selected DEPs that upregulated in ADSC EVs compared to UC-MSC EVs.

To further investigate the mechanism underlying the observed efficacy of EVs in the skin cell models, we conducted a comprehensive characterization of the functional biocomponents present in both ADSC and UC-MSC EVs, with a particular focus on their miRNA and protein content. Although miRNA constitute only a minor portion of the RNA cargo within EVs, they are known to play a significant role in regulating target cell functions (Bi et al., 2022). Our analysis identified 736 miRNAs in ADSC EVs and 1,137 miRNAs in UC-MSC EVs. Of these, 686 miRNAs were common to both EV types (Figure 6a). The ten most abundantly expressed miRNAs in each EV type were determined based on their relative expression levels (Figure 6b). Several highly expressed miRNAs - specifically Let-7a-5p, Let-7c-5p, Let-7f-5p, miR-16-5p, and miR-199b-3p - were shared between the two EV populations. To further explore the potential functions mediated by these shared miRNAs, a clustering analysis on their 662 common miRNAs was performed to predict their putative target genes in human cells. These predicted target genes were then cross-referenced with the DEGs identified earlier, resulting in a subset of 33 genes shown in Figure 6c. Notably, among these 33 genes, several are associated with key skin functions previously described in Section 3.4, including genes involved in epidermal keratinization and development, as well as cell-cell adhesion, such as CLDN17 and CDH2. Additionally, the list includes genes implicated in immune response and inflammation, such as IL6 and CXCL8.

Proteomic profiling of ADSC and UC-MSC EVs showed that 1,054 proteins were present in both types of EVs (Figure 6d). Nearly one-fifth of the 1,054 common identified proteins participate in intracellular signaling cascades or receptor binding, and a comparable proportion of the identified proteins are collectively implicated in inflammatory responses, lipid and vesicle-mediated transport, translation, and cytoskeletal organization. Approximately 10% of detected proteins in EVs are associated with cell adhesion, protein folding, macromolecular complex assembly, and organic substances. A smaller subset of 5% are involved in the regulation of cell homeostasis, proteolysis, apoptosis, protein localization, and similar functions. Proteins were ranked based on relative abundance, with several proteins present in both the top 10 of ADSC and UC-MSC EVs, including alpha-2-macroglobulin (A2M), complement component 3 (C3), actin (ACTB), immunoglobulin heavy chain (IGHM), and albumin (ALB). Compared to UC-MSC EVs, ADSC EVs displayed 73 differentially expressed proteins, including significantly higher expression of proteins of COL1A2, COL3A1, TIMP3, and others (Figure 6f). These proteins are linked to pathways associated with skin development, extracellular matrix (ECM) organization, cell-matrix adhesion, which could account for the difference underlying the observed bioactivities.

## 4 Discussion

Extracellular vesicles have garnered considerable attention due to their potential to modulate immune responses, mitigate cellular senescence, stimulate angiogenesis, promote collagen synthesis, and decrease epigenetic age (Boulestreau et al., 2020; Sanz-Ros et al., 2022; Zhao H. et al., 2023). The primary objective of this work was to explore the potential of EVs previously linked to regenerative behaviors for their ability to impact biomarkers associated with skin aging in a 3D reconstructed skin model. In this work, 3D *in vitro* skin models were employed to evaluate the efficacy of MSC-EVs, as such models offer a more physiologically relevant approach compared to conventional 2D cell assays, while being able to bridge mechanistic understanding gaps that human studies cannot address. However, it is essential to acknowledge that *in vitro* models cannot fully replicate the complexity of skin, including its diverse cell types and immune processes. Therefore, the key findings pertaining to efficacy and mechanisms must undergo validation through clinical studies, accompanied by thorough safety and regulatory assessments.

Extracellular vesicles from a variety of sources are currently being studied ranging from disease biomarkers to tissue repair therapeutics (Tesovnik et al., 2020; Nagelkerke et al., 2021; Vyas et al., 2023). Given that there are few published reports of clinical efficacy, the present study was designed to focus on EVs from ADSCs and UC-MSCs, two cellular sources previously studied for regenerative behaviors in 2D and pre-clinical tests. Consistent with prior studies (Ha et al., 2020; Pomatto et al., 2021), ADSC EVs and UC-MSC EVs both increased fibroblast proliferation in standard 2D assays conducted herein. The proliferative response exhibited a plateau rather than exhibiting a linear concentration-dependent effect. As EV signaling is complex, and EVs are capable of interacting with cells through a variety of mechanisms (including internalization, membrane fusion, and receptor binding), it is difficult to identify the reason behind the lack of a clear concentration-dependent response in the specific experiments conducted in this study. As such 2D assays with a single cell-type are not completely predictive of how EVs would perform in 3D, multi-cell systems, the T-skin™ model, which offers the ability to explore impact on epidermal maturation, differentiation, and potential crosstalk with an underlying model dermis (Michelet et al., 2012; Bataillon et al., 2019), was leveraged for in-depth mechanistic investigation. Compared to untreated groups, administration of ADSC and UC-MSC EVs to the culture media in the T-skin™ model significantly enhanced epidermal thickness, keratinocyte proliferation, collagen IV expression in the DEJ, and fibrillin 1 expression in the dermis. Collectively, these results emphasize the multi-faceted potential of EVs to impact many cell types and layers that may contribute to an overall improvement in skin health. In addition, transcriptomics analysis was conducted on the T-skin™ models treated with or without EVs, showing EV regulation of genes related with skin regeneration and anti-inflammation. This finding is consistent with previous studies demonstrating that MSC EVs can decrease the expression of pro-inflammatory cytokines (e.g., IL-6, IL-1β, TNF-α) which are also representative senescence-associated secretory phenotype (SASP) components in cell senescence (Li et al., 2023), while increasing the expression of anti-inflammatory cytokines (e.g., IL-10) to facilitate skin repair (Janockova et al., 2021; Marofi et al., 2021). While some earlier studies attempted to highlight the differences between EVs from various cell and tissue sources (Pomatto et al., 2021), the observation from the current study also identifies outcomes in which both ADSC and UC-MSC EVs had similar impact on skin (e.g., increased epidermal thickness).

The transcriptomic and proteomic profiles of the EVs offer potential insights into the commonality as well as distinctive functionalities of EVs derived from different sources, and effectively illustrate the complexity of EV cargo. Bioinformatic analysis of miRNA and protein cargo of MSC-EVs from distinct tissue sources revealed both a significant overlap in their content, as well as differences in the molecules selectively presented in ADSC versus UC-MSC EVs. It is possible that many of the observed outcomes from the 2D and 3D cell models were driven, in part, by a subset of miRNA and protein cargo, instead of any specific one. For instance, many miRNAs and proteins identified in both ADSC EVs and UC-MSC EVs are associated with increased cell proliferation. MiR-21 enhances keratinocyte and fibroblast proliferation through upregulation of the PI3K/AKT signaling pathway, promoting MMP-9 secretion (Zhang et al., 2021) and downregulating PTEN and SPRY1 (Hu et al., 2018). MiR-135a and miR-126-3p promote fibroblast proliferation by downregulating LATS2 and downregulating PIK3R2 (Ma et al., 2022), respectively (Ju and Liu, 2023). The presence of STAT3 protein in MSC-EVs has been reported to enhance the proliferation and migration of diabetic wound fibroblasts and augment endothelial angiogenesis (Shabbir et al., 2015). Furthermore, cadherins and integrins, major adhesion molecules, were identified in both EV populations and are crucial for fundamental cellular processes such as adhesion and proliferation (DiPersio et al., 2016; Qiu et al., 2019). In addition to the impact on cell proliferation, bulk RNA-sequencing of the T-skin™ models also revealed a potential anti-inflammatory benefit to the ADSC and UC-MSC EVs. Proteins like PDL1 and TGF-β, both present in ADSC and UC-MSC EVs, have been implicated in the immunomodulatory function of MSC-EVs, leading to the elevated production of anti-inflammatory cytokines (Mokarizadeh et al., 2012). In addition, miRNAs were also identified in both types of MSC EVs. For example, miR-203 reduces NAD + depletion, improves mitochondrial function and decreases cell senescence (Zhao L. et al., 2023). MiR-let-7c-5p, miR-214-3p and miR-125b-5p identified in both EVs tested here, were previously identified in EVs from young mouse ADSCs that improved frailty and health span of aged mice (Sanz-Ros et al., 2022). Another miRNA found in both ADSC and UC-MSC EVs, miR-17, could extend mouse lifespan by inhibiting senescence signaling mediated by MKP7 according to previous studies (Du et al., 2014; Kinser and Pincus, 2020). These findings offer a potential explanation for the observed efficacy of MSC EVs in regulating skin regeneration-related biomarkers *in vitro*. At the same time, the apparent redundancy in activators and inhibitors of multiple biological pathways highlights the challenge in establishing component specific mechanism for the biological effect of EVs, and rather encourages a holistic evaluation approach to focus on the downstream cellular response rather than EV composition.

Herein, we were able to cross-reference EV miRNA and protein profiles, to the transcriptomic response of the T-skin™ models (Figure 6c). It was apparent that downregulated genes in the T-skin™ models, such as TP63, CCL4, CCL4L2, CXCL8, and IL6, were typical pro-inflammatory transcription factors (Kubo et al., 2021), mediators (Estevao et al., 2021; Cambier et al., 2023; Tsai et al., 2023), and receptors (Hirano, 2021) that could have been inhibited by the corresponding miRNAs from the EVs. Interestingly, we also found that APOB, TAS2R3, and CYP26B1, individually being aging biomarker (Richardson et al., 2021), bitter taste receptor (Tuzim and Korolczuk, 2021), and retinoic acid catabolic enzymes (Pavez Loriè et al., 2009), were also known targets of the miRNAs that were inhibited in the skin model. However, we noted several genes that were known targets of the miRNAs in the evaluated EVs and were upregulated in the conditions of the study. The function of these upregulated genes found in our study, including CLDN17, SPRR, CHAC1, KRT75, CDH2, are reported enriched in the regulation of differentiation/proliferation in prior studies (Gibbs et al., 1993; Li et al., 2020). Collectively, these identified proteins and miRNAs, along with their reported functionalities, provide a basis for understanding the regulatory effects of MSC EVs on skin cells observed *in vitro* in this study. While it is tempting to attribute biological efficacy to a specific population of miRNAs or proteins, further analysis is needed to effectively prove the delivery, processing, and causative impact of such cargo. In this study, the transcriptomics results from the 3D models reflect the collective response of both epidermal and dermal compartments, rather than cell specific signatures. This limitation could be addressed in future studies by separating the tissue layers or through techniques like single-cell RNA sequencing. Additionally, it is unknown whether functional miRNAs and proteins work independently or synergistically when transferred from MSC-EVs to target cells.

Cell source, culture conditions, harvesting period, and isolation methods were all reported to impact the structure and function of EVs (Almeria et al., 2022; Zeng et al., 2022; Wang et al., 2023). Human-derived EVs, including UC-MSC and ADSC EVs, also face challenges regarding donor-dependent variability and the technical complexity of scaling standardized production. Various techniques, including preconditioning the parent cells, 3D culture using a bioreactor, physical or chemical stimulation, genetic manipulation, and exposure to hypoxia, are under exploration to generate unique EVs (Marofi et al., 2021). Moreover, the choice of isolation methods, such as size exclusion chromatography and ultracentrifugation, can influence the ratio of recovery and protein amount in EVs. The cargoes of produced EVs are also influenced by the culture environment and protocol, necessitating comprehensive characterization of EV cargo to examine its regeneration effects. The MISEV2023 guidelines provide best practice guidance for EV studies, including identification and characterization. In this study, EVs were procured by a single supplier based on their standardized production procedure, and characterization was conducted following MISEV2023 guidelines. The presence of EVs was confirmed prior to any 2D or 3D efficacy evaluation by quantifying particle concentration, visualizing particles, and confirming the presence of associated biomarkers via Western blot analysis. With that said, the results from this study should not be interpreted as if all EVs derived from ADSCs or UC-MSCs would be expected to behave in the same way. Prior to making such assertions, a similar testing protocol to that implemented herein should be followed on the final EV material to understand their signature as it relates to skin aging biomarkers.

To date, numerous efforts have been undertaken to expedite the functional mapping of EVs, emphasizing the crucial need for a standardized evaluation approach. With European legislation prohibiting animal testing for cosmetic ingredients, the demand for reproducible and high-throughput alternative *in vitro* methods has become imperative. In comparison to monolayer cell cultures, 3D human skin equivalents derived from human cells offer a more faithful representation of cellular organization and function. EPISKIN® has adeptly adapted its process for mass-producing a full-thickness skin model, namely, T-Skin™, in accordance with ISO9001 standards, ensuring a high level of reproducibility. This reliable full-thickness model has proven valuable for both research applications and routine testing. In our investigations, the studies conducted on both 2D cells and 3D T-skin™ models underscored the promising potential of EVs in regulating skin-related biomarkers linked to regeneration and aging. Looking ahead, these *in vitro* models present a transformative opportunity for the cosmetic industry, paving the way for next-generation active ingredients in anti-aging, regenerative, and protective skincare formulations. Nevertheless, the pivotal findings related to efficacy and underlying mechanisms necessitate validation through clinical studies, complemented by thorough safety and regulatory assessments.

## 5 Conclusion

In this study, 3D reconstructed skin models were utilized to explore how EVs previously linked to regenerative benefit impact skin biomarkers associated with aging. Specifically, EVs from both ADSCs and UC-MSCs were evaluated through a multi-faceted approach, combining characterization, functional validation, bulk transcriptomics, and proteomic and miRNA composition analysis, unveiling their profound capability to actively drive key aspects of skin regeneration. The findings presented here highlight the ability of 3D reconstructed skin models to decipher the role of EVs in promoting essential cellular functions such as keratinocyte proliferation, reinforcement of the dermal ECM, and enhancement of collagen IV production at the DEJ. Furthermore, integrating bulk transcriptomics with miRNA profiling provided mechanistic insights, elucidating the complex molecular regulatory networks modulated by these EVs. Collectively, this study expanded on established pro-regenerative potential of EVs by demonstrating their potential to regulate skin regeneration related biomarkers in 3D reconstructed skin models. Looking forward, future investigation is worthwhile on EV production, understanding cargo synergy, and translating these insights into clinical applications.

## Data Availability

The datasets presented in this study can be found in the article/Supplementary Material, with further inquiries can be directed to the corresponding authors.
