# Supplementary material for: Extracellular vesicles modulate skin aging biomarkers in a 3D reconstructed full-thickness skin model
Source: Front Cell Dev Biol. 2026 Mar 6;14:1784998. doi: 10.3389/fcell.2026.1784998 (PMC13003219; doi:10.3389/fcell.2026.1784998)
Supplement: Supplementary file 1 [file Supplementaryfile1.docx]

Supplementary Material

Extracellular Vesicles Modulate Skin Aging Biomarkers in a 3D Reconstructed Full-Thickness Skin Model

Yao TENG^1^, Elias BOU SAMRA^2^, Sarah GIRARDEAU-HUBERT^2^, Richard J. BETTS^3^, Franck JUCHAUX^2^, Xavier MARAT^2^, Benedicte FALLOU^4^, Lingyan ZHONG^1^, Rodrigo De VECCHI^2^, Nan HUANG^1^, Qian ZHENG^5^, Yu GAO^1^*, Daniel C ROY^5^, and Ping WANG^1^*

^1^Advanced Research, L'Oréal Research & Innovation, Shanghai, China;

^2^Advanced Research, L'Oréal Research & Innovation, Aulnay-sous-Bois, France;

^3^Advanced Research, L'Oréal Research & Innovation, Singapore;

^4^EPISKIN, L'Oréal Research & Innovation, Lyon, France;

^5^Advanced Research, L'Oréal Research & Innovation, Clark, New Jersey, United States;

*** Correspondence:**grace.gao@loreal.com

ping.wang@loreal.com

# Supplementary Figures

**Figure S1 Filaggrin expression in skin model with or without EV treatment**


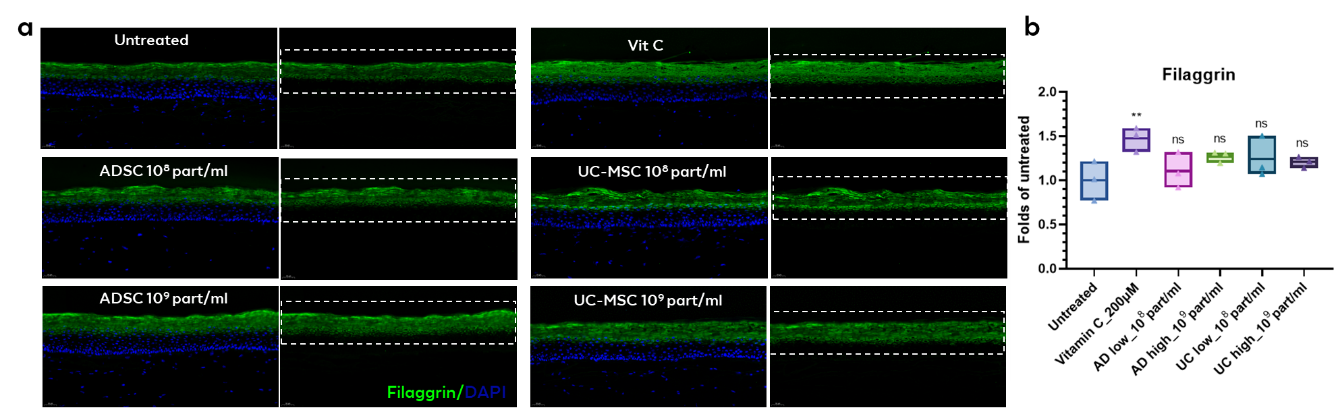


**Supplementary Figure 1.** EV effects on ageing biomarkers in 3D reconstructed skin model. (a) Immunofluorescent images for filaggrin in skin model sections with different EV treatment conditions and (b) quantitative results for filaggrin expression. One-way ANOVA, *p<0.05, **p<0.01, ***p<0.001. n=3, representative of 3 independent experiments.

**Figure S2 Venn plot of DEGs regulated by EV treatment at high and low concentrations**


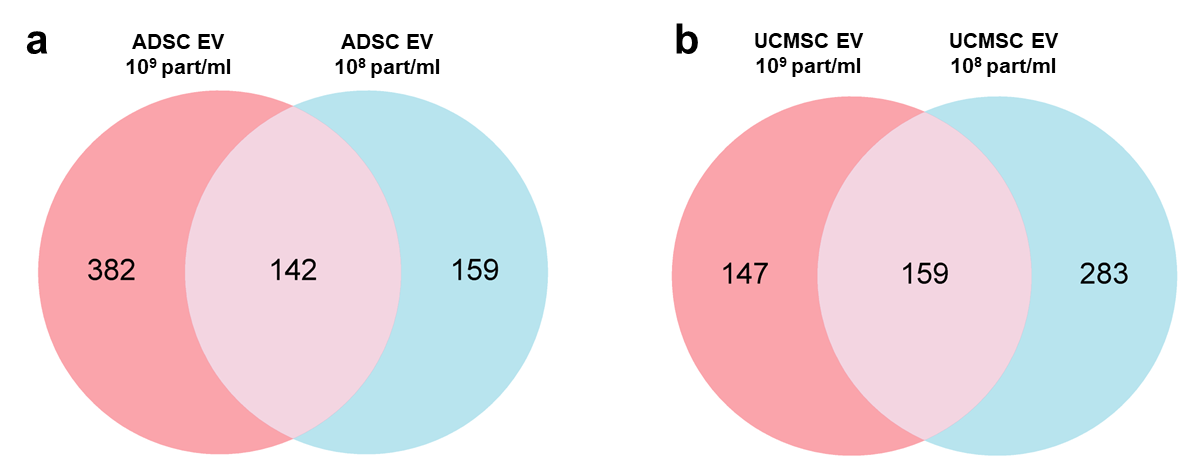


**Supplementary Figure 2.** DEG number regulated by (a) ADSC EVs and (b) UC-MSC EVs at high and low concentrations was shown in the Venn plot.
